# Robotics and AI for Teleoperation, Tele-Assessment, and Tele-Training for Surgery in the Era of COVID-19: Existing Challenges, and Future Vision

**DOI:** 10.3389/frobt.2021.610677

**Published:** 2021-04-14

**Authors:** Navid Feizi, Mahdi Tavakoli, Rajni V. Patel, S. Farokh Atashzar

**Affiliations:** ^1^ Canadian Surgical Technologies and Advanced Robotics (CSTAR), London Health Sciences Centre, and School of Biomedical Engineering, University of Western Ontario, London, ON, Canada; ^2^ Department of Electrical and Computer Engineering, University of Alberta, Edmonton, AB, Canada; ^3^ Department of Electrical and Computer Engineering, University of Western Ontario, London, ON, Canada; ^4^ Department of Surgery, University of Western Ontario, London, ON, Canada; ^5^ Department of Electrical and Computer Engineering, New York University, New York, NY, United States; ^6^ Department of Mechanical and Aerospace Engineering, New York University, New York, NY, United States

**Keywords:** COVID-19, robotics, surgery, teleoperation, tele-examination, tele-training

## Abstract

The unprecedented shock caused by the COVID-19 pandemic has severely influenced the delivery of regular healthcare services. Most non-urgent medical activities, including elective surgeries, have been paused to mitigate the risk of infection and to dedicate medical resources to managing the pandemic. In this regard, not only surgeries are substantially influenced, but also pre- and post-operative assessment of patients and training for surgical procedures have been significantly impacted due to the pandemic. Many countries are planning a phased reopening, which includes the resumption of some surgical procedures. However, it is not clear how the reopening safe-practice guidelines will impact the quality of healthcare delivery. This perspective article evaluates the use of robotics and AI in 1) robotics-assisted surgery, 2) tele-examination of patients for pre- and post-surgery, and 3) tele-training for surgical procedures. Surgeons interact with a large number of staff and patients on a daily basis. Thus, the risk of infection transmission between them raises concerns. In addition, pre- and post-operative assessment also raises concerns about increasing the risk of disease transmission, in particular, since many patients may have other underlying conditions, which can increase their chances of mortality due to the virus. The pandemic has also limited the time and access that trainee surgeons have for training in the OR and/or in the presence of an expert. In this article, we describe existing challenges and possible solutions and suggest future research directions that may be relevant for robotics and AI in addressing the three tasks mentioned above.

## Introduction

The novel coronavirus has been declared a public health emergency of international concern by WHO in Jan 2020 (WHO, 2020). By the time of writing this paper, all countries are affected by the pandemic. The unprecedented shock wave of the virus spread has impacted regular health care service delivery. The extreme pressure on healthcare systems has exceeded capacity, and managing the pandemic has become a global issue that has drastically influenced most aspects of the healthcare system. The performance of surgeries (most of which are categorized as elective surgeries), training for surgery, and assessments for surgery are aspects that have been significantly impacted.

Due to the chance of false negatives in the pre-surgery COVID-19 testing of patients, all patients have to be treated as suspect cases. Dealing with infected or suspected patients requires precautions such as consideration for anesthesiologists (Willer et al., 2020), limitation of staff exposure to patients, and wearing PPE, which poses difficulties to operating theatre (Kumar et al., 2020). However, these procedures cannot guarantee the safety of staff and patients. Moreover, since hospital staff is in contact with several people each day, cross-infection through staff should also be considered.

New regulations have recommended a moratorium on elective surgery to avoid virus spread in hospitals by minimizing personal interactions and expenditure of medical resources for infected patients who need intensive care. Deferring elective surgeries is based on opinions and has secondary consequences. Progression of the disease continues when the patient is waiting for surgery. This has a substantial impact on the life quality of patients (Fu et al., 2020), results in higher treatment costs (Reyes et al., 2019), and may cause unexpected death (Zafar, 2020). On the other hand, elective surgeries are not optional and must be performed eventually. Thus, there may be a need for performing a deferred surgery during the COVID-19 time frame. Moreover, catching up with the 2 million backlogged elective surgeries worldwide each week will impose a huge burden on the healthcare system when elective surgeries resume (Szklarski, 2020).

This unprecedented scenario not only has affected surgeries, but has also influenced surgery-related activities profoundly. Surgical education has been affected adversely by the pandemic. There has been a gradual reopening of activities, including schools, but with the anticipated rise in the rate of infection it is anticipated that there will be some levels of shut down again in this sector. Trainees are banished from wards, and residents have lost their access to practical OR training (Ferrario et al., 2020; Ferrel and Ryan, 2020). In addition, emergency and non-deferable surgeries are being done by senior surgeons without the presence of trainees to reduce operation time and risk of complications, and mitigate the risk of residents’ exposure to COVID-19 (Bernardi et al., 2020). This situation has imposed mental anxiety and has slowed down the learning curve of residents and medical students, who will be needed in catching-up with deferred surgeries in the future (Ahmed et al., 2020).

Moreover, going into hospitals for pre- and post-surgery assessments is also a safety concern during the pandemic. There is always an infection risk for any minute that a non-COVID patient spends in a hospital. Consequently, hospitals try to discharge patients as soon as possible to reduce the risk of infection. Besides, the closure of medical offices has disturbed pre- and post-surgery assessments (Scaravonati et al., 2020).

Since there is no widely approved or sufficiently tested vaccine, there is a possible chance of second and third global waves in the Fall and Winter, and continuing lockdown regulations imposes an intolerable burden on the healthcare system. Several countries are therefore planning for a reopening guideline to resume safe delivery of surgical services (Dattani et al., 2020). However, it is not clear how the reopening phase will affect the quality of healthcare delivery and how the above-mentioned tasks should be performed safely while there is a lack of a clinically approved therapy for COVID-19. This perspective paper proposes robotics and artificial intelligence (AI) as a solution for the three above-mentioned tasks and investigates potential opportunities in the area to address the mentioned problems.

## Robotics-Assisted Surgery

Minimally invasive surgery (MIS) has demonstrated superiority over open surgery. Less amount of blood loss, and shorter recovery and hospital stay are the main reasons for the preference of MIS when it is possible. Meanwhile, robotics-assisted MIS (RAMIS) has evolved and shown superhuman capabilities for teleoperation and has found its place in MIS. Teleoperation offers surgeons an ergonomically operating posture (Ballantyne, 2002), provides them with more dexterity than conventional laparoscopy (Moorthy et al., 2004), enhances the accuracy of motion beyond surgeons’ natural ability, etc. Besides these benefits, the main virtue that distinguishes teleoperation in the COVID-19 era is providing the ability to separate the surgeon’s console (leader robot) from the patient robot (follower robot) while keeping them connected through a communication interface (Challacombe et al., 2003).

Telesurgery affords physical separation of the surgeon from the patient, in a separate room avoiding bilateral infection transfer, which can be life-threatening. In addition, the number of bedside staff in RAMIS is less than in open surgery (Kimmig et al., 2020). This provides the safety of the patient and operating room by reducing inter-personal contacts to the lowest level possible. This performance has been shown experimentally. In the United States, a CorPath robotic intervention arm has been used in coronary intervention on a COVID patient to provide safety of the personnel (Tabaza et al., 2020).

Dealing with an infected or suspected patient requires a maximal level of protection (Liang, 2020). The physical disturbance caused by this level of protection has a negative influence on surgical performance. On the other hand, the COVID-19 situation increases the surgeon’s mental stress, which critically affects the surgeon’s performance. Elevated stress levels could likely be due to the fear of contracting the virus or spreading it to patients and the surgeon’s family (Tan et al., 2020). Studies have shown that depression, anxiety, insomnia, and stress have increased, especially among front-line healthcare providers during the pandemic (Lin et al., 2020). High stress levels may result in inappropriate responses, such as poor decision making and impaired psychomotor performance (Wetzel et al., 2006; Arora et al., 2010). The elevated psychological stress levels among healthcare providers may sustain even one year after the outbreak as it happened in 2004 with SARS (Lee et al., 2007). Not only telesurgery reduces surgeon’s stress by providing better ergonomics during surgery (Berguer and Smith, 2006), but also, during the COVID era, robotics-assisted surgery significantly reduces stress levels by providing higher infection protection through physical distancing between the patient and the surgeon; also, it reduces the number of needed medical staff to be present in close proximity to the patient and each other during prolonged surgeries (which can increase the possibility of infection transfer between a patient and staff and between staff members). It should be highlighted that robotics assisted surgery does not make the aforementioned interactions zero as there is always the need for some format of interaction between a patient and staff. However, it reduces the duration and the number of interactions.

Recently, the concept of semi-autonomous and autonomous surgery has attracted a great deal of interest thanks to advancements in the area of machine intelligence especially when combined with computer vision (Moawad et al., 2020). When compared with teleoperated robotic surgery, AI-based autonomous and semi-autonomous robotic systems has not been fully exploited in the literature as these are newer topics of the field. In the language of surgery project, it has been shown that combining AI with teleoperation can provide a semi-automated system that can recognize and perform tasks automatically when there is a pre-trained model for the recognized task, allowing for faster and high accuracy procedures (Bohren et al., 2011). Semi-autonomous robots have been used for orthopedic surgery, such as MAKOplasty, when preoperative images are fused with intraoperative information to provide surgeons with an augmented sensorimotor capability through production of dynamic virtual fixture in time and space. More recently, fully-autonomous robotic surgery has been discussed in medical robotic communities and preliminary experiments on *ex-vivo* tissue have shown promising results. The performance and accuracy of semi-autonomous surgical robots have been proved clinically (Hampp et al., 2019). For example, the MAKO (Stryker, 2020) and NAVIO (Smith and Nephew, 2020) robots guide the surgeon in joint arthroplasty semi-autonomously and prevent excessive bone loss. This guarantees proper bone preparation and precise implantation. However, autonomous surgical robots, despite their great accuracy in comparison to manual procedures (Shademan et al., 2016), are still in the non-clinical development phase. In the context of remote operation, the use of autonomous robots can provide a higher degree of separation while providing some additional accuracy through processing of multimodal intraoperative information. However, this is a technologically challenging field which should be investigated to provide more autonomy regarding management of surgery during a crisis such as COVID-19.

The other benefit of RAMIS in the COVID-19 era is that it increases the availability of intensive care unit (ICU) beds. The smaller incision for RAMIS shortens patient’s recovery time and hospital stay. This allows hospitals to dedicate more ICU beds to critically ill cases while handling surgeries. There is however a shortcoming in terms of the OR time usage for RAMIS as a result of the extra setting-up time and longer procedure times (Heemskerk et al., 2007; Cho et al., 2016; Lindfors et al., 2018). Nonetheless, a shorter post-surgery hospital stay is of paramount importance and outweighs the longer OR time, notably in the COVID-19 era. Moreover, deploying AI in robotic surgery has been shown to decrease soft tissue damage and consequently decrease recovery time (Wall and Krummel, 2020).

Due to abdominal pressure in laparoscopic surgery, there are some concerns about the possibility of aerosolization of viral particles and contamination through surgery smoke in laparoscopic surgery (Schwarz and Tuech, 2020; Van den Eynde et al., 2020). Although these methods of infection are not completely proved for COVID-19 yet, safety regulations should be considered to prevent possible infections. It should be noted that surgical smoke is also released in open surgeries; however, in RAMIS, it is easier to handle the smoke trapped in the patient’s body. Safety precautions to prevent these issues are 1) lowering the electrocautery power to reduce the amount of smoke production (Mottrie, 2020); 2) smoke evacuation and abdominal deflation through ultra-low penetrating air (ULPA) filter (Kimmig et al., 2020); and 3) reducing abdominal pressure to the lowest possible. RAMIS surgeries are feasible to perform with lower abdominal pressure than conventional laparoscopic surgery (Kimmig et al., 2020). To summarize, RAMIS is safer than MIS and open surgery in terms of contamination through aerosolization of viral particles for bedside staff.

Telerobotic surgical systems have solved several issues associated with conventional MIS and also provided the surgeon with new capabilities. These features are 1) depth perception; 2) dexterity enhancement; 3) improved accuracy; 4) better hand-eye coordination; and 5) and multiple tools delivery through a single incision (Atashzar and Patel, 2018). Moreover, in teleoperation, information and operation data can be saved and used for training purposes both for AI supervision and training of novice surgeons (Zemmar et al., 2020). The problem of degraded haptic feedback in conventional laparoscopy has not been solved yet. Better tracking accuracy and improved surgical performances have been achieved using the haptic feedback in RAMIS (Talasaz et al., 2014), (Currie et al., 2017). Related to this, the lack of haptic feedback increases the risk of tissue damage due to large unintentional forces. Other modalities of feedback such as visual force feedback of the tool (Tavakoli et al., 2006), a tactile sensor and tactile ultrasound (tactUS) instrument for palpation and tumor localization (Trejos et al., 2009; Naidu et al., 2017a; Naidu et al., 2017b), and skin stretch feedback (Schorr et al., 2013) are influential in robotic surgery, but none of them can completely make up for the absence of haptic feedback. Thus, enabling telerobotic surgical systems with force sensing and force reflecting modules is of high importance, which increases the quality of teleoperated surgery (Talasaz and Patel, 2013; Talasaz et al., 2017). A machine learning algorithm has been deployed to estimate the elongation of suture from knot type, initial suture length, and surgical thread type data, and visual feedback has been used to warn the surgeon of the risk of suture breakage (Dai et al., 2019). In particular, considering a larger number of surgeries that can benefit from teleoperated procedures using robots, during the era of COVID, addressing this challenge should be accelerated. This topic has seen ongoing research, and unfortunately, the current trend does not show a promise of an upcoming solution. With improving technology for haptic feedback, this can result in a major advance in the performance of teleoperated surgeries on a larger scale and can enlarge the domain of surgeries, which can be conducted teleoperatively, helping with the management of the current concerns regarding infection transfer during surgery in the time of COVID.

There are two characteristics associated with a good haptic teleoperation system: transparency and stability. In the last three decades, a significant amount of research has been done on developing a transparent control architecture. Four-Channel Lawrence (FCL) was proposed as the first transparent teleoperation system (Lawrence, 1993). It was modified to simpler architectures (Hashtrudi-Zaad and Salcudean, 2001), (Hashtrudi-zaad and Salcudean, 2002). Atashzar et al. proposed a simplified two-channel modified-ELFC (M-ELFC) architecture that provides a high degree of transparency (Atashzar et al., 2012). To deal with the stability issue, three categories of passivity-based controllers have been proposed: 1) the Wave Variable approach (Aziminejad et al., 2008); 2) Time Domain Passivity Approach (TDPA) (Ryu et al., 2010); and 3) Small-gain approach (Atashzar et al., 2017a). Both techniques stabilize the system; however, stabilization comes at the cost of compromising transparency. Considerable research has been done to improve the performance of teleoperation (Artigas et al., 2010; Chawda and Omalley, 2015; Atashzar et al., 2017b; Panzirsch et al., 2019; Singh et al., 2019), but the proposed stabilization methods are still far from ideal. The discussion above clarifies some of the technical challenges creating obstacles to realizing high-fidelity haptics-enabled teleoperated surgery. The potential of RAMIS in resolving the surgical issues caused by COVID-19 is calling for an accelerated trend of research and development, extending the performance of teleoperated surgical robotic systems for allowing more benefit of this technology in reducing the burden on the healthcare system during the pandemic and similar crises in the future.

Remark: AI has been extensively developed in the last decade and has revolutionized many industries. However, the application of AI in surgical procedures requires a significant amount of adaptation and consideration. Robotic surgery can take advantage of AI in the COVID era from three aspects; 1) increasing accuracy and reducing the risk of failure by providing shared and full autonomy in simple tasks (Rabinovich et al., 2020; Wall and Krummel, 2020); 2) allowing physical distancing by changing the surgeon’s role from executive and continuous control to supervisory and intermittent control; and 3) increasing the average number of surgical procedures, which will be required to address the backlogged surgeries caused by the shutdown of elective surgeries over a long period of time, thereby reducing the load on surgeons and allowing after-hour surgeries (Zemmar et al., 2020).

## Tele-Examination of Patients

Preoperative examination for surgery preplanning and post-operative patient examination in the recovery time is another matter of concern in the COVID-19 era. In-person visits increase the risk of virus contraction for the patient and the surgeon. Keeping personal interactions as low as possible is the key factor in dealing with the pandemic.

Post-operative examinations may include patient’s assessment at home and ICU. Telepresence robots that are made for telehealthcare purposes allow physicians to interact with patients, and monitor patients’ vital signs without the physical presence of the surgeon in the ICU (Laniel et al., 2017). These systems have been used in Italy at COVID-19 patients’ bedside in the ICU (Bogue, 2020; Pullella, 2020). A similar robot has been used in Israel to communicate with quarantined patients (Marks, 2020). In terms of home healthcare, messages, phone, and video calls have been used for post-operative examination. It has been shown that telepresence robots could provide a stronger feeling of a person to person interaction for both users, in comparison to video and phone calls, and both physicians and patients have expressed satisfaction (Tavakoli et al., 2020), (Becevic et al., 2015).

Preoperative examinations have also been done with AI- and robotics-enabled telehealth, but applications are limited due to the lack of physical exams and the need for clinical imaging. However, it has been shown that for some specific conditions, diagnosis via telemedicine could be as accurate as an in-person diagnosis when examination through telemedicine is feasible subject to limitations (Asiri et al., 2018). For example, AI can be used for digital triage to direct patients to the most appropriate medical center based on the resources and their condition before they show up in emergency rooms (Lai et al., 2020). As another example, it has been shown that blood draw and injections can be done with portable robots using AI more accurately and faster than a manual procedure (Zemmar et al., 2020). Another example is the telerobotic system that has been used in China to perform cardiac and lung ultrasound on a COVID patient (Wang et al., 2020). These systems can help safeguard patients and staff by reducing the need for patient referral to hospital and physical distancing.

Robotics and AI have taken a step in the development of tele-examination of patients during pre- and post-operative phases. However, there is still room for adding new capabilities to tele-healthcare robots in order to lower the need for in-person examinations or patient referrals to hospital. Besides robots, focusing on smartphone-based or computer-based tele-examination systems would be useful because of their widespread use.

## Tele-Training of Surgeons

The outbreak of COVID-19 has severely affected surgical training procedures. The most significant components of surgical training are comprised of theoretical, pre-clinical, and hands-on clinical training, but the lockdown caused by the pandemic has severely limited the opportunities for students and residents to acquire surgical training (Puliatti et al., 2020), (Bernardi et al., 2020). High-quality and intense healthcare support, which would be needed during and after COVID-19, requires precise training. Although some schools are in a gradual reopening phase, there would be some level of shut down again with the next wave of the pandemic.

In such extraordinary conditions, online learning, teleconferences, and webinars can be of benefit with regard to surgical education and fill the gap with regard to theoretical training issues (Dedeilia et al., 2020). The benefits of these online learning technologies have been shown prior to the COVID-19 pandemic.

On the other hand, robotics and AI could improve the quality of pre-clinical training. Pre-clinical training is conventionally performed through dry or wet lab practices. The use of robotic simulators based on virtual reality (VR) systems has shown a significant improvement in novice surgeons’ skills (Tergas et al., 2013). Hands-on-Surgical Training (HoST) provided by augmented reality (AR), and dual-user teleoperated system with virtual fixtures are more advanced simulators that help the novice surgeon to navigate using haptics-enabled cues outside the OR (Shahbazi et al., 2013; Kumar et al., 2015). Xperience Team Trainer developed by Mimic Simulations allows teamwork training in the OR at the pre-clinical stage (Mimics, 2020). This technology provides simultaneous training for the novice surgeon and the bedside assistant to improve coordination between the surgeon and assistant.

The preservation of acquired skills is another important issue in the COVID time. Surgical skills including motor and cognitive skills decay when a surgeon goes through a long period of time without using the acquired skills (Perez et al., 2013). Simulation-based medical education may fill the gap in surgical practice and prevent the loss of surgical skills during a lockdown (Higgins et al., 2020). In addition, AI can be employed to interpret the data collected from simulations for surgeons’ skill evaluation (Winkler-Schwartz et al., 2019).

Because the above-mentioned technologies provide high-quality training while keeping social distancing, they could be part of the solution for the educational gap in the COVID-19 era. An active line of research and development that can be accelerated would be to design and develop small, inexpensive, and portable sensorized robotic modules connected to cloud-based virtual reality surgical environments. A large number of trainee surgeons could then continue their hands-on practice/training when access to training facilities is significantly restricted. This is critical because sensorimotor learning is a continual process in the human brain, and a long pause before getting to the agency level can drastically result in fading of sensorimotor skills.

Theoretical and pre-clinical training may guide students to pass the cognitive phase of learning; however, the integration phase, which gives them appropriate motor skills to perform surgery, requires performing surgery under the supervision of an expert in the OR (Choi et al., 2020). Residents would have very limited access to this form of training due to cancelation of elective surgery (Imielski, 2020) or requirements of social distancing. For telesurgery which is more challenging for residents to perform than open surgery and requires specific training, a viable solution that can be achieved using existing systems can be developed using hand-over-hand haptic-enabled tele-training (realized by multilateral teleoperation systems). This would not only allow novice surgeons to perform surgery from a safe distance, but also give them the opportunity to be supervised by an expert at the same time (Shahbazi et al., 2018a; Shahbazi et al., 2018b). The dual console teleoperation system format shares the control of the operation between the expert and the trainee. Incorporating haptics-enabled feedback would then provide the trainee with real-time force feedback. This format of telesurgery training gives the resident experience through supervised surgery without jeopardizing the safety of the patient or the resident during the constraints imposed by COVID-19. Furthermore, these multilateral tele-training systems could also be set up to evaluate the motor skills of trainees based on their performance.

## Discussion and Conclusion

The novel coronavirus has challenged the healthcare system across the globe. Social distancing has become a new normal and may remain for a significant length of time especially as a result of the lack of vaccine and treatment for a critical period of time. This has deeply impacted surgeries and surgically related activities which may revolutionize how future healthcare systems function. Canceling elective surgeries was an efficient policy to curb the spread of the virus; nevertheless, keeping to this plan could have a detrimental effect on the health of patients and the healthcare system. Currently, governments are working on reopening guidelines. In this unprecedented situation, robotics and AI could play an important role in the safe delivery of surgical services through the use of telesurgery, tele-examination, and tele-training environments. A summary of the existing technologies and required features is given in Table 1.

**TABLE 1:** Existing technologies and required features in telesurgery, tele-examination, and tele-training.

	Current existing technologies translated into practice	Required missing features for performance improvement
Telesurgery	•Unidirectional teleoperation. pros: better ergonomy; physical separation; less bedside staff; shorter hospital stay; less abdominal pressure; simpler surgical smoke handling; automated data recording.cons: lack of force feedback in the loop; limited types of surgeries. •Visual and other modalities of force feedback. pros: better diagnosis in teleoperation and less tissue damage while avoiding instability.cons: not as effective as direct haptic feedback.	•Transparent direct haptic feedback. •Increased capability to include more types of surgeries. • Reduce the cost of robots to increase accessibility. • Development of shared autonomy between surgeons and robots. • Automating simple tasks using AI to augment the performance, fluency and consistency of the surgery while reducing the need for interpersonal interaction.
Tele-examination	•Telemedicine systems through voice and video conferencing. pros: no need for hospital attendance; minimizing the risk for patients to come into contact with the source of infection; minimizing the need for traveling to clinics enhancing the accessibility; allowing for more-frequent visits; better digital platform for tracking records and conditions.cons: not as effective as in-person examination in many cases due to limitations on conducting physical exams.	•Automated triage using AI. •Portable examination system for pre-and post-surgery. • Telepresence robots in ICU and patients’ houses for tele-physical examination. • Advanced automated wearable systems for tracking patient's vital signs and physical ability.
Tele-training	•Online learning systems. pros: following up the theoretical aspects of surgical training during lockdown; minimize the need for in-person attendance.cons: lack of experimental training. •VR robotic surgery simulation systems. pros: effective experimental training for students while minimizing the risk of making mistakes in actual surgery and minimizing students risk of infection. cons: not as effective as actual wet lab training.	•Hands-on-Surgical Training through dual-console telesurgery systems. •A portable hands-on robotic module to provide consistency in surgical training. • Accurate surgical skill evaluation system using AI and actual saved telesurgery data.

Regarding teleoperated robotic surgery, it should be noted that although there is a wide range of benefit for both patients and the surgeons, there still exist a spectrum of challenges which are open topics for research and development. Regarding benefits, in the context of laparoscopic surgery, it can be mentioned that besides reduced operation time, reduced blood loss, increased accuracy, and reduced recovery time, there are additional benefits that are more pronounced during the pandemic, including reduced time and frequency of interpersonal interaction between surgical staff, reduced number of staff, reduced interaction between patients and staff, all to reduce the risk of infection transfer and increase the safety of surgical procedures (Kimmig et al., 2020; Tavakoli et al., 2020). It should be noted that the current state of telesurgery and robotics-assisted surgery are advanced for abdominal surgery; however, for some categories such as orthopedic surgery, teleoperation has not been considered as a robust option. During the pandemic, any technology that reduces the duration of surgery directly or indirectly (for example, by increasing the accuracy which reduces the need for readjustments) can significantly reduce the chances of infection transmission. This is critical since, in general, surgeons operate on many patients in a short time, which can increase the risk of infection even between patients indirectly through their surgeon.

However, it should be noted that there is a wide range of challenges which have not been addressed yet, especially in the context of teleoperated surgery, and these for the future direction of research. One of the major challenges is the stability and transparency of force-feedback teleoperated robotic systems (Aziminejad et al., 2008; Ryu et al., 2010; Atashzar et al., 2017a). Due to the concerns of safety the existing commercialized telerobotic surgical systems, such as the da Vinci surgical system, do not enable force reflection, even though it is known that force reflection can significantly increase the quality of surgery by providing a much higher situational awareness for surgeons. Although a number of stabilizers and control algorithms have been reported in the literature, the existing algorithms result in deviation of motion tracking and force reflection, which reduces the accuracy of surgery and is often not acceptable (Artigas et al., 2010; Chawda and Omalley, 2015; Atashzar et al., 2017b; Panzirsch et al., 2019; Singh et al., 2019). Besides stability, instrumentation is another challenge. Attaching inexpensive, disposable, biocompatible, and miniaturizable force sensors to surgical tools for measuring multidimensional forces for reflection through a teleoperation medium is a major instrumentation challenge and an open line of research (Atashzar and Patel, 2018). Technologies such as optical force sensors are promising options and are the front line of research in this regard.

In addition, the introduction of AI in telesurgery is a new field of research and development which has attracted a great deal of interest in order to enable parts of surgical tasks to be automated, thereby reducing some cognitive and physical burden for the surgical team with the potential for reducing the operation time, increasing accuracy and reducing the number of needed staff in the operating room. The accuracy resulting from using AI in industrial applications has been shown; however, more research is required to prove its performance and build up confidence in the medical area (Wall and Krummel, 2020). Dealing with soft tissue is the main challenge when involving AI in the context of robotic and telerobotic surgery.

Regarding tele-examination, telepresence robots have been effective in improving post-operative patient-surgeon interactions and monitoring patient’s vital signs, mostly in ICUs. However, due to limitations, effective solutions for detailed pre- and post-operative tele-examination of patients have not been proposed in the literature. One of the main challenges in this area is the development of portable sensorized robots for detailed remote monitoring of patient’s signs. On the other hand, AI would be particularly useful in automating tele-examination devices to reduce the need for in-person pre- and post-operative examinations.

As for tele-training, simulation-based training systems using AR and HoST have provided a context for pre-clinical training while ensuring the safety of trainees and experts. In addition, simulation-based training can be effective in ensuring skill levels of surgeons in the presence of long periods of surgical inactivity. However, there are open areas for research in this field. Hand-over-hand training using multilateral teleoperation is one of the future research areas that can profoundly improve the quality of clinical surgical tele-training. The stability of delayed multilateral teleoperation and effective methods for sharing control between an expert and a trainee are directions for future researches (Shahbazi et al., 2018a; Shahbazi et al., 2018b). Besides hand-over-hand training, employment of AI for surgical skill training and assessment are open research areas.

In this perspective article, we have provided our opinions on some existing technologies which can be adopted rapidly to help with the current unprecedented situation and have given a perspective of the technologies required in hospitals. The intention in writing this article has been to initiate discussions between researchers, policymakers, and stakeholders to further investigate the use of robotic, telerobotic and AI-based solutions in a framework for enhancing the performance of surgery, surgical training, post-operative treatment, and monitoring under the severe restrictions imposed by COVID-19. The vision and opinions presented in this article are based on an extensive review of the literature concerning approaches through which Robotics and AI can play a significant role.

## Data Availability

The original contributions presented in the study are included in the article/Supplementary Material, further inquiries can be directed to the corresponding author.
